# SARS-CoV-2 surveillance in US wastewater: Leading indicators and data variability analysis in 2023–2024

**DOI:** 10.1371/journal.pone.0313927

**Published:** 2024-11-18

**Authors:** Hannes Schenk, Wolfgang Rauch, Alessandro Zulli, Alexandria B. Boehm

**Affiliations:** 1 Department of Civil & Environmental Engineering, School of Engineering and Doerr School of Sustainability, Stanford University, Stanford, CA, United States of America; 2 Unit of Environmental Engineering, University of Innsbruck, Innsbruck, Austria; Chulalongkorn University Faculty of Medicine and King Chulalongkorn Memorial Hospital, THAILAND

## Abstract

Wastewater-Based Epidemiology (WBE) has become a powerful tool for assessing disease occurrence in communities. This study investigates the coronavirus disease 2019 (COVID-19) epidemic in the United States during 2023–2024 using wastewater data from 189 wastewater treatment plants in 40 states and the District of Columbia. Severe acute respiratory syndrome coronavirus 2 (SARS-CoV-2) and pepper-mild mottle virus normalized SARS-CoV-2 RNA concentration data were compared with COVID-19 hospitalization admission data at both national and state levels. We further investigate temporal features in wastewater viral RNA abundance, with peak timing and cross-correlation lag analyses indicating that wastewater SARS-CoV-2 RNA concentrations precede hospitalization admissions by 2 to 12 days. Lastly, we demonstrate that wastewater treatment plant size has a significant effect on the variability of measured SARS-CoV-2 RNA concentrations. This study highlights the effectiveness of WBE as a non-invasive, timely and resource-efficient disease monitoring strategy, especially in the context of declining COVID-19 clinical reporting.

## 1. Introduction

On 30 January 2020, the World Health Organization (WHO) declared the coronavirus disease 2019 (COVID-19) outbreak a public health emergency of international concern [1]. Three years and three months later, the WHO declared the end of the public health emergency, despite severe acute respiratory syndrome coronavirus-2 (SARS-CoV-2) infections remaining a leading cause of death worldwide and in the United States (US). Even with the availability of vaccines and therapeutic treatments in the US, SARS-CoV-2 was responsible for a reported 49,931 deaths in 2023, highlighting the need to understand COVID-19 disease burden to inform public health policies [2].

While clinical data remain the standard for tracking disease burden, maintaining testing on a large scale is resource intensive, fails to detect asymptomatic cases and relies on the compliance of the public [3]. Wastewater-based epidemiology (WBE) provides a complementary monitoring option that helps fill knowledge gaps such as undetected community spread, asymptomatic cases, and the lag in clinical reporting [4–6]. Human shedding of pathogens and chemicals into wastewater provides an important source of information on the health of the entire community living in a catchment area [7]. As an established surveillance method, WBE contributed to the management of the COVID-19 pandemic early on. Medema *et al*. [8], Ahmed *et al*. [9] among others [10–14] laid out the foundational work for SARS-CoV-2 surveillance using wastewater. While clinical testing efforts have decreased, WBE remains a central technology in monitoring SARS-CoV-2 in the population, as well as increasing in use for the detection of other pathogens [15–18].

Timely epidemiological data are crucial for assessing infectious disease outbreaks and implementing the necessary public health interventions. Both clinical testing data and hospital admission data correlate strongly to viral RNA concentrations in wastewater, with wastewater leading both clinical testing and hospitalization data [14, 19–22]. WBE as an early warning system has been discussed in literature thoroughly [23, 24]. A streamlined process of logistics, sample analysis and data reporting are critical to leverage the temporal advantages of WBE. Understanding the lead times of WBE data is critical for the construction of forecasting models.

In this study the 2023–2024 COVID-19 epidemic in the US is investigated by analyzing longitudinal measurements of SARS-CoV-2 RNA in wastewater from 189 wastewater treatment plants (WWTPs) throughout the US. The data are aggregated on several spatial levels to compare to data on COVID-19 hospitalizations in 10 states. This paper’s novelty lies in its extensive dataset from WWTPs across the US, providing a comprehensive nationwide analysis of the 2023–2024 post-emergency period. We then investigate leading or lagging behavior by comparing peak timings and computing maximum cross-correlation coefficients. Lastly, we demonstrate that SARS-CoV-2 RNA concentration variability is a function of WWTP size, offering new insights into the influence of WWTP size on the underlying data volatility characteristics.

## 2. Methods

### 2.1. Viral RNA quantification and data characterization

For this study, wastewater data and hospitalization data are analyzed. COVID-19 hospitalization data are publicly available from the Centers for Disease Control and Prevention (CDC) [2]. State-aggregated, daily hospitalization data consists of data on COVID-19 occupancy and admission numbers. The wastewater data used in this study were retrieved through the nucleic acid extraction of settled solids from WWTPs nationwide. In June 2024 a total of 189 treatment plants were monitoring SARS-CoV-2 RNA in 40 different states throughout the US using a standardized approach by a single laboratory. Wastewater composite samples are collected with a sample frequency of 2 to 3 times per week for most plants, while some plants collect samples up to 7 times per week. Settled solids were extracted after dewatering by centrifugation at 24,000 x g for 30 minutes [25]. Solids were resuspended in DNA/RNA shield to a concentration of 75 mg/mL. Bovine coronavirus (BCoV) was used as a positive recovery control in all samples. Extraction was performed using a Chemagic Viral DNA/RNA 300 kit H96 in conjunction with a Perkin Elmer Chemagic 360 instrument (Chemagic #CMG-1033-S). Inhibitor removal was performed using a Zymo OneStep-96 PCR Inhibitor removal kit (Zymo Research #D6035). Extraction negative controls and positive controls were extracted during the same run. SARS-CoV-2 digital droplet RT-PCR was performed using primers and probes previously described [25]. BCoV and pepper-mild mottle virus were quantified in a duplex assay in each sample as controls. Each sample was run in 6 to 10 replicate wells and merged before analysis [26]. In the study period between 1^st^ of May 2023 and 1^st^ of June 2024, a total of 29,364 daily samples were examined. Within this timeframe the number of monitored plants remained relatively stable. Readers are directed to the Data Descriptor for a full description of the SARS-CoV-2 measurements methods [27].

**Fig 1:**
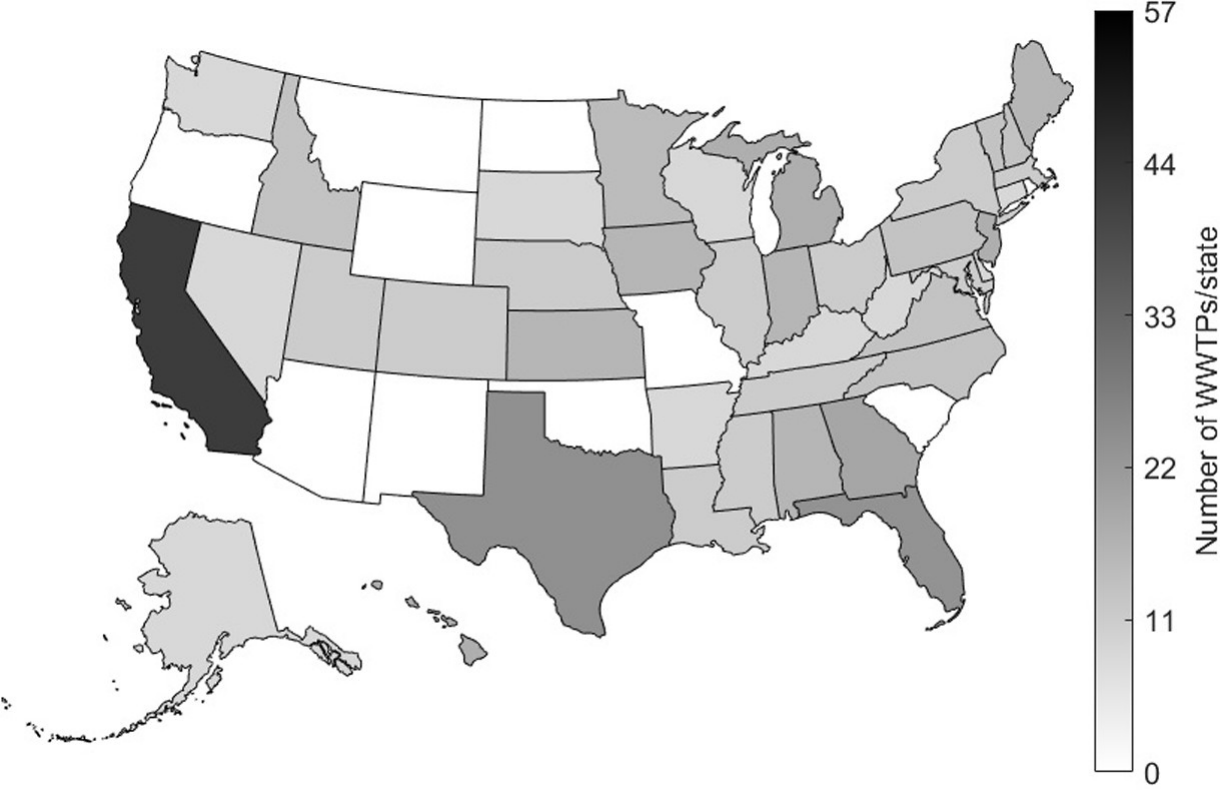
Map of the US. shading indicates the number of WWTPs contributing to WBE in each state.

Fig 1 illustrates a map of the US, highlighting the number of WWTPs participating in SARS-CoV-2 RNA wastewater surveillance for the project. California, Texas, and Florida are the states with the highest number of contributing WWTPs, with 57, 14, and 13 plants respectively. Overall, 40 states have at least one WWTP monitoring SARS-CoV-2. Table 1 provides a detailed list of the states in the US, including the number of WWTPs contributing to the study. It also includes the total population served and the percentage of population coverage in each state.

**Table 1:** Number of WWTPs by state and percentage of population covered.

State	#WWTPs	Pop. served/10^3^	Pop. coverage %	State	#WWTPs	Pop. served/10^3^	Pop. coverage %
CA	57	20,511	52.6	UT	2	715	20.9
TX	14	2,425	7.9	TN	2	700	9.8
FL	13	3,650	16.1	OH	2	539	4.6
GA	8	1,109	10.1	LA	2	383	8.4
NJ	6	1,882	20.3	NE	2	300	15.2
HI	6	858	59.8	IL	2	149	32.7
MI	6	482	4.8	MD	2	145	1.2
AL	5	627	12.3	NY	2	120	2.3
IN	5	322	4.7	CO	2	60	0.6
KS	5	267	9.1	MS	2	53	1.0
ME	5	185	13.3	NV	1	990	1.8
IA	5	129	4.0	KY	1	423	31.0
MN	4	326	5.7	AK	1	220	9.4
PA	3	361	2.8	CT	1	140	3.9
ID	3	345	17.6	WV	1	100	5.6
NC	3	167	3.1	WI	1	44	0.7
VA	3	153	1.8	SD	1	20	2.2
NH	3	79	5.6	AR	1	15	0.5
VT	3	56	8.7	DE	1	13	1.3
MA	2	2,650	37.8	WA	1	10	0.1

### 2.2. Data pretreatment

SARS-CoV-2 RNA (SC2) and pepper mild mottle virus RNA (PMMoV) concentrations were measured with digital droplet RT-PCR and reported as gene copies per gram dry weight. PMMoV-RNA is shed by humans in great abundance following the consumption of bell pepper and other pepper products [28]. Dividing SC2 by PMMoV RNA concentrations compensates for the diversity of fecal strength of waste stream. This concept follows the mass balance model that relates concentrations of SARS-CoV-2 RNA in wastewater solids to incident infections of individuals in the sewershed [29]. The PMMoV normalized SC2 concentration was computed as follows:

$$SC2_{PMMoV}=\frac{SC2}{PMMoV}$$ (1)

Before the spatial aggregation of WBE data, the data were examined for outliers. Wastewater data are marked by random and systematic errors. Random errors are immanent to the technique of WBE and caused by heterogeneities in the environmental sample and processes that affect concentrations in the sample; these can be difficult to reduce. Systematic errors are caused by a failure in the measurement process. With the outlier removal approach in this work, systematic errors are targeted. Concentrations larger than 3 standard deviations above the log_10_ transformed mean of the entire dataset (n = 29,364) are discarded. It is acknowledged that all samples represent real data, but real data is prone to have errors. Without outlier detection, the analysis has no mechanism of protection against errors.

The analyses outlined in the following subsections have been performed on both raw SC2 concentrations and PMMoV normalized data. We present the results of the PMMoV normalized analyses, considering it is a standard procedure in the field. The conclusions remain unchanged whether PMMoV normalization is applied or not (data not shown). S1 Fig in the S1 File compares raw SC2 RNA and PMMoV normalized SC2 RNA concentrations.

### 2.3. Spatial data aggregation

In order to compare wastewater data with hospitalization levels on a state by state basis (or on national scale), the SC2 concentration measurements were spatially aggregated. The spatial aggregation for WBE data in a state was performed by computing weighted daily averages of all WWTPs that provided data at a given date in that state, where the weighting factor is the population size that each plant serves. This computation results in a representative daily average of the SC2 circulation in the state, where the size of the plant was taken into consideration accordingly. The state aggregated daily weighted averages are calculated by

$$SC2_{PMMoV,S}(d)=\left(\sum_{i=1}^{P}SC2_{PMMoV,i}(d)*pop_{i}\right)/\sum_{i=1}^{P}pop_{i}$$ (2)

where the summation over the *P* indicates the plants in state S and *pop*_i_ denotes the population served by plant *i*. Analogous to the spatial aggregation on a state level, national weighted daily averages are computed by utilizing all available plants in the US. To obtain gapless time-series for the temporal analysis of the data, linear interpolation was performed if no datapoint was available at a given day after spatial aggregation.

Spatial aggregation is an essential computational step to ensure that WBE data can be accurately compared with other epidemiological data of a different spatial scale. Without proper weighting, aggregating WBE data from various WWTPs would lead to biased results, as smaller plants would exert disproportionate influence, while larger plants would be underrepresented. To address this, a population-weighted approach was employed, ensuring that each WWTP’s contribution is proportional to the population it serves. While PMMoV normalization accounts for variations within individual catchment areas, it may not adequately address the significant disparities in plant sizes.

### 2.4. Temporal analysis

The temporal features of PMMoV RNA-normalized SC2 concentrations (SC2_PMMoV_) in wastewater are investigated in this study and compared on a state by state basis to hospitalization admission. The association between WBE data and hospitalization admission was determined using two approaches. First, cross-correlation function analysis (CCF) and second by examining the peaks in SC2_PMMoV_ over time. Waves are periodic surges or peaks in the concentration of SC2_PMMoV_ over time. Furthermore, Spearman correlation *r* was examined to outline the quantitative relation between the time series.

Peak timing in time series provides a good reference point for comparison. The COVID-19 epidemic in the US in the latter half of 2023 was characterized by a wave peaking in early fall, followed by a larger wave peaking in January 2024. In this work the peak timings for hospitalization admission and SC2_PMMoV_ in wastewater are compared relative to one another on a state by state basis. Peaks are determined by locating the highest values of the 7-day moving mean of the SC2_PMMoV_ concentrations in wastewater and hospitalization admission time-series. The peaks are determined for both occurring waves, where the 1^st^ of November is the date of separation between first and second wave. This date was chosen by visual inspection of the data and allows for a good separation of the two peaks for all states. The average time differential $\bar{\Delta t}$ was than calculated by averaging the difference between peak occurrences of the two peaks for each state. $\bar{\Delta t}$ was calculated by

$$\bar{\Delta t_{s}}=\frac{\Delta ta+\Delta ta}{2}$$ (3)

where Δ*t*_*peak*_1,*S*_ and Δ*t*_*peak*_2,*S*_ denote the time difference in days between the peaks of hospitalization admission and SC2_PMMoV_ concentrations for the two respective waves one and two. The subscript *S* denotes the state. The hospitalization peak date *t*_hosp_ was subtracted from the wastewater peak date *t*_ww_, so that negative days signify an earlier peak date in wastewater

$$\Delta t_{peak,S}=t_{ww}-t_{hosp}$$ (4)

The reliability of the results is influenced by the abundance of WBE data available in each state. For this reason, the analysis in this study primarily focuses on the 10 states with the highest population coverage (from Table 1, CA, FL, NJ, HI, ID, MA, UT, NE, IL, KY). All analyses are performed with MATLAB 2023b, The MathWorks Inc.

### 2.5. Data dispersion analysis

SC2 and PMMoV RNA concentrations in wastewater are characterized by substantial amounts of variability. Herein, data dispersion and variability characteristics are examined to quantify WBE data attributes. Data variability was explored for different sizes of WWTPs, where the proxy for plant size is given by the number of populations that each plant serves. The population served by the plants varies significantly, with the smallest plant serving approximately 5,000 individuals and the largest, a plant in Los Angeles, California, serving 4 million individuals. This analysis aims to investigate whether there are significant differences in data properties between small and large plants. It is hypothesized that differences in concentration variability may be observed due to the substantial variation in plant size, spanning three orders of magnitude.

To investigate the potential differences in concentration behavior between small and large wastewater treatment plants, the WBE dataset was partitioned into five groups. The partitioning regime was determined by quantile intervals of the population served. Data groups corresponding to the five quantile intervals are denoted as Q_0-0.2_, Q_0.2–0.4_, …, Q_0.8–1_ (from smallest 20% of plants to largest 20% of plants). Table 2 outlines the quantile intervals and data partitioning regime used for this analysis. The grouping in the described manner is designed to partition plants into groups that have similar sizes.

**Table 2:** Data partitioning into quantile ranges of plant sizes.

Quantile ranges	Population served	#WWTPs	SC2 data points
Q_0-0.2_	5,000–30,000	42	6278
Q_0.2–0.4_	30,001–64,000	34	4697
Q_0.4–0.6_	64,001–102,125	38	5632
Q_0.6–0.8_	102,126–227,238	38	6324
Q_0.8–1_	227,239–400,0000	38	6433

For each of the five data groups standard deviation (SD) and interquartile-ranges (IQR) are computed of the log10 SC2 concentrations. This enables a comparison of the degree of variability as a function of plant size. To test that the five data groups stem from different statistical populations, two-sided Wilcoxon rank sum tests are performed between adjacent data groups. The data dispersion analysis was carried out on raw SC2 (not PMMoV normalized) RNA concentrations. This ensures that data variability changes based on plant size are not driven by effects in PMMoV, but SC2 RNA concentrations.

## 3. Results

**Fig 2:**
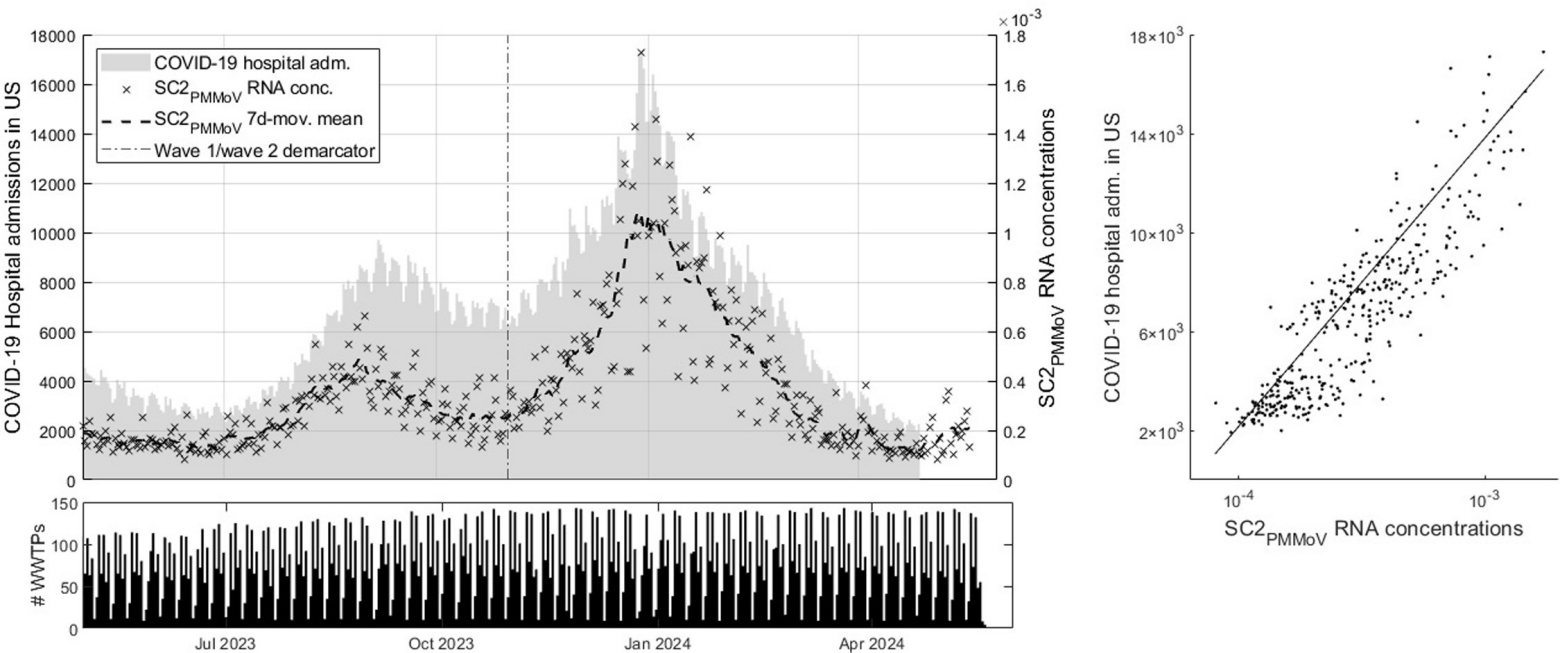
National aggregation of SC2_PMMoV_ and COVID-19 hospitalization admissions (top left), number of WWTPs measured per day (bottom) and OLS regression between the datasets.

The COVID-19 epidemic in the timeframe May 2023 to June 2024 was characterized by waves in SC2 RNA concentrations, similar to previous years [30]. This is shown in Fig 2, plotting national aggregated daily SC2_PMMoV_ concentrations (and its 7-day moving average) along with hospitalization admissions in the US. This figure outlines the general development of the epidemic in the US in the studied timeframe. The two peaks are visually evident in the national aggregated wastewater data. Reporting of SC2 hospitalization data was discontinued from the beginning of May 2024 and therefore truncated in Fig 2. The bottom bar chart outlines the number of WWTPs that are monitored at a particular day. On the right of the figure, a scatter plot depicts WBE and hospitalization admission data with a simple ordinary least squares (OLS) regression line.

**Fig 3:**
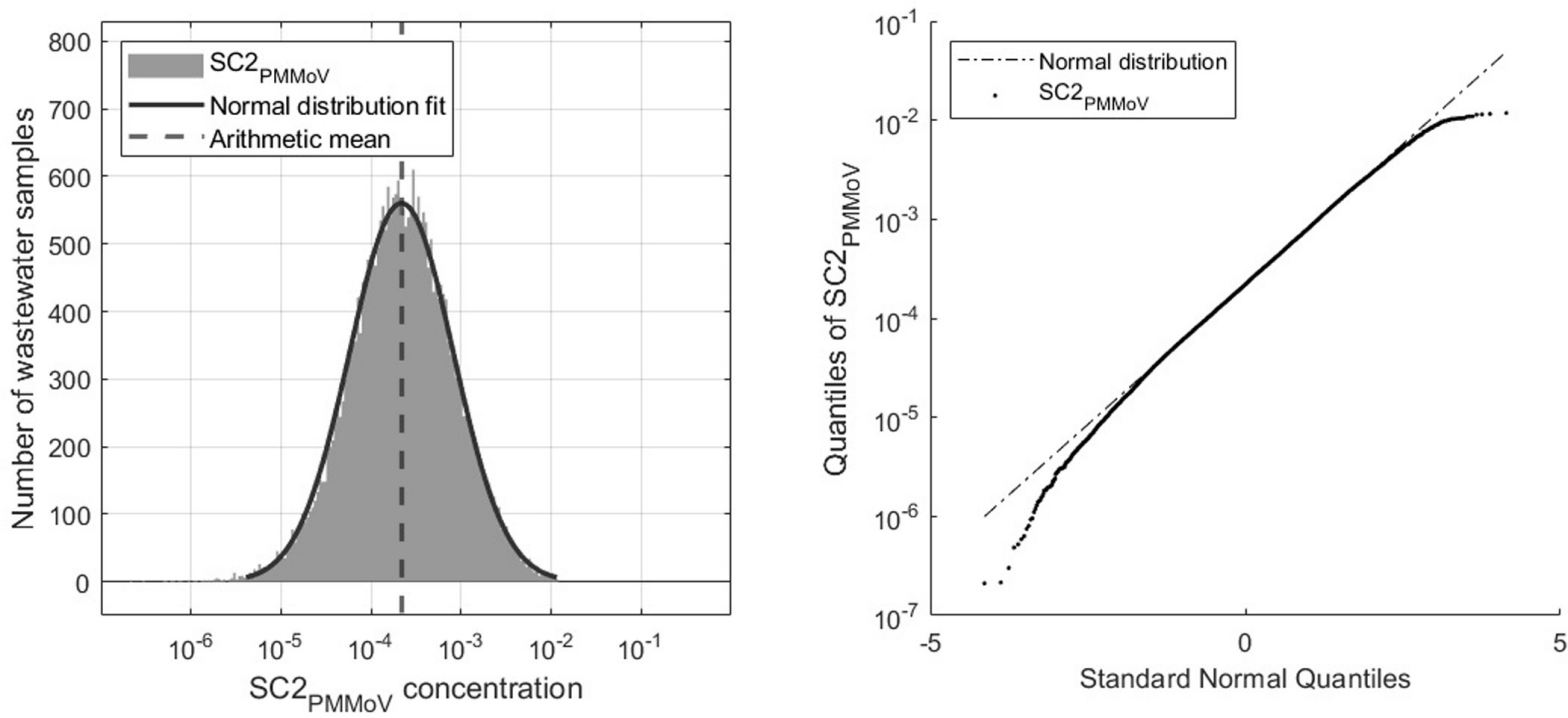
SC2_PMMoV_ data distribution and QQ-plot.

Fig 3 displays the histogram of all SC2_PMMoV_ data in the timeframe May 2023 to June 2024. Values above 3 standard deviations above the mean are discarded as systematic errors (27 data points out of 29,364). The mean normalized concentration was 0.00052 and the resulting outlier threshold 0.012 (SC2_PMMoV_ concentrations are unitless, because it is a fraction of concentrations). Three standard deviations above the mean on the log_10_ transformed data corresponds to a 24-fold higher concentration on linear scale in relation to the mean of the data.

In addition to the histogram on Fig 3, a normal distribution fit is computed to outline the resemblance of the SC2_PMMoV_ data with a log-normal distribution. To test the log_10_ transformed data for normality, Shapiro-Wilk and Kolmogorov-Smirnov tests are performed with a 5% significance level each. Both tests reject the null hypothesis and suggest that the data are not normally distributed. Compared to an ideal log-normal distribution, the measured data are characterized by a fat tail on the left. Unlike the right side of the distribution, the left side is not truncated with a lower bound for outlier removal. Low/very low concentration values are not considered outliers. On the right graph of Fig 3, the quantile-quantile (QQ) plot is depicted. It can be seen that both tails of the distribution deviate from the normal line. The data are characterized by a slight negative skew (skewness = -0.16).

### 3.1. Temporal analysis results

In epidemiological surveillance, early detection and rapid information processing are critical. Temporal features of WBE SC2 monitoring, such as peak timing, cross-correlation lag and temporal trends are analyzed. While other epidemic waves of diseases like influenza or respiratory syncytial virus are characterized by clear onset/offset dates, SC2 has been persistently circulating in the population since its outbreak in 2020 [31]. Resulting from the lack of a clear onset/offset condition in the case of SC2 RNA concentrations, peak timing by state was investigated in this work.

**Fig 4:**
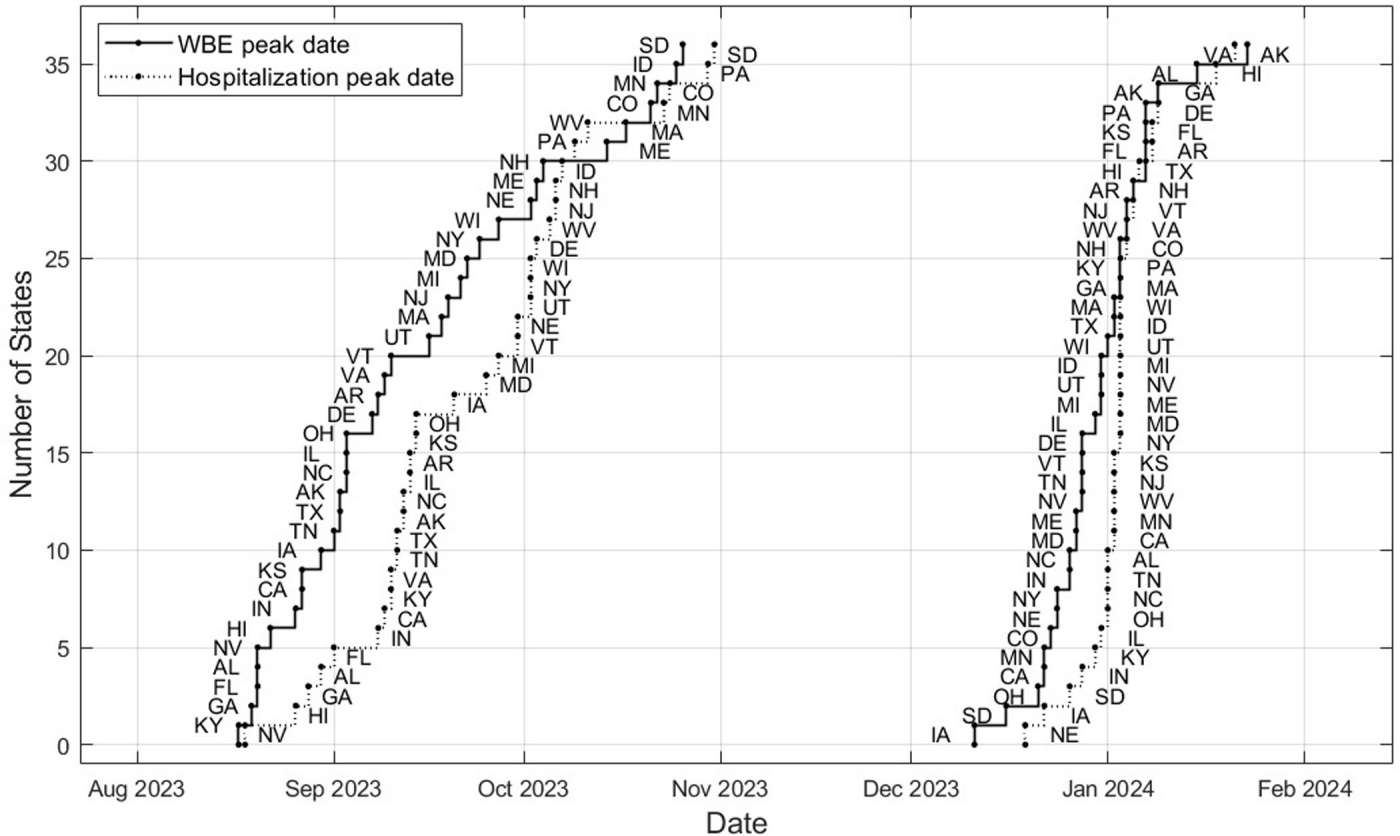
Cumulative number of states by peak occurrence, WBE and hospitalization admission. Abbreviation for each state is provided next to its data point.

The COVID-19 epidemic in the US in the timeframe 2023–2024 was characterized by two waves. The peak of the first wave was characterized by a lower magnitude in SC2_PMMoV_ concentrations and hospitalization and occurred for most states in September 2023. The second wave occurred around January 1^st^ 2024. Fig 4 shows the cumulative occurrence of peaks in wastewater and hospitalization data for each state and for both waves.

It can be seen in Fig 4 that the WBE peak generally occurs earlier than the hospitalization peak. The peak timings between hospitalization admission and SC2_PMMoV_ concentrations decrease between the first and second wave. The difference between the median dates of hospitalization peaks and SC2 peaks was 9 days for the first wave and 4 days for the second wave. Spearman rank correlation of the order in which the states occur was 0.91 for the first wave and 0.56 for the second wave. This means that generally the order of occurrence of peaks is respected between the two data sets, especially in the first wave. The first wave occurs earliest in the Southeastern region including the states Kentucky, Georgia, Florida and Alabama, followed by Nevada, Hawaii and California among others. The second wave peaks earliest in midwestern states including Iowa, South Dakota, Minnesota, accompanied by Oklahoma, California and Nevada.

**Fig 5:**
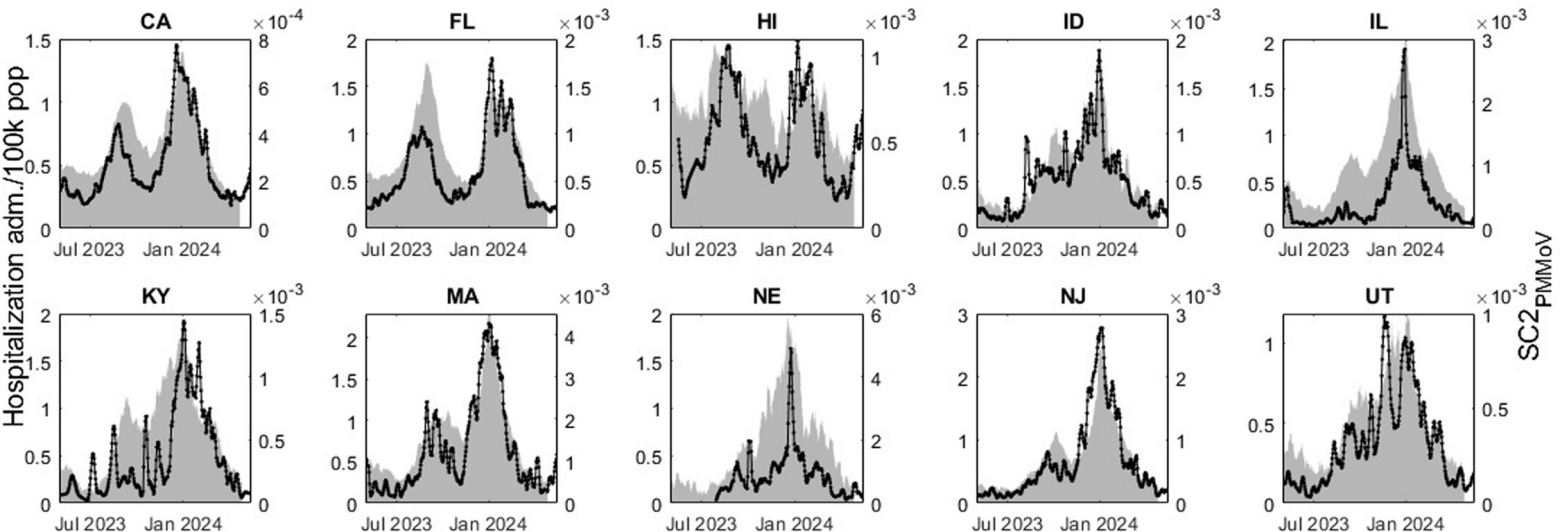
State aggregated SC2_PMMoV_ (black line) and hospitalization admission (gray bars).

Fig 5 displays the state aggregated SC2_PMMoV_ concentrations and superimposed hospitalization admission per 100k population (gray bars) for the 10 states with the highest WBE coverage in the US. SC2_PMMoV_ is shown on the left axis on log scale and hospitalization is shown on the right axis on linear scale.

Table 3 outlines the results of the temporal analysis. CCF lag between the time series (state aggregated SC2_PMMoV_ and hospitalization admission) and the time differential $\bar{\Delta t}$ of the relative peak occurrence are listed. Negative values of CCF lag and $\bar{\Delta t}$ indicate that the WBE peak occurred before the hospitalization peak. Furthermore, Spearman correlation *r* values are listed as a comparative analysis between SC2 hospitalization admission and wastewater data for the 10 states with the highest WBE coverage.

**Table 3:** SC2_PMMoV_ and hospitalization temporal quantitative feature comparison by state. Negative lag values indicate a time lead in wastewater over hospitalizations.

State	$\bar{\Delta t}$ (d)	CCF lag	Spearman *r*
CA	-12	-3	0.88
FL	-6.5	-5	0.86
HI	-7.5	-10	0.62
NJ	-7.5	-4	0.93
ID	7.5	-3	0.86
MA	-12	-4	0.83
NE	3.5	4	0.85
UT	-9.5	-5	0.86
AK	-11	-9	0.67
NV	-2	-2	0.76

SC2 in wastewater leads hospitalization admission in 8 out of 10 states, following the results of peak timing $\bar{\Delta t}$. For the CCF lag, 9 out of 10 states show this characteristic. A median time lead of 4 and 7.5 days was observed for CCF lag and $\bar{\Delta t}$ respectively among the 10 states with the highest WBE population coverage. The correlation metrics *r* and *R*^2^ suggest a close agreement between hospitalization admission and SC2_PMMoV_ in wastewater (median *r* = 0.85).

### 3.2. Data dispersion results

Differences in data dispersion characteristics for different plant sizes are observed. To examine the influence of plant size, the wastewater data are partitioned into 5 groups. The partitioning is governed by the quantiles of the population served by each plant and carried out as described in section 2.5.

**Fig 6:**
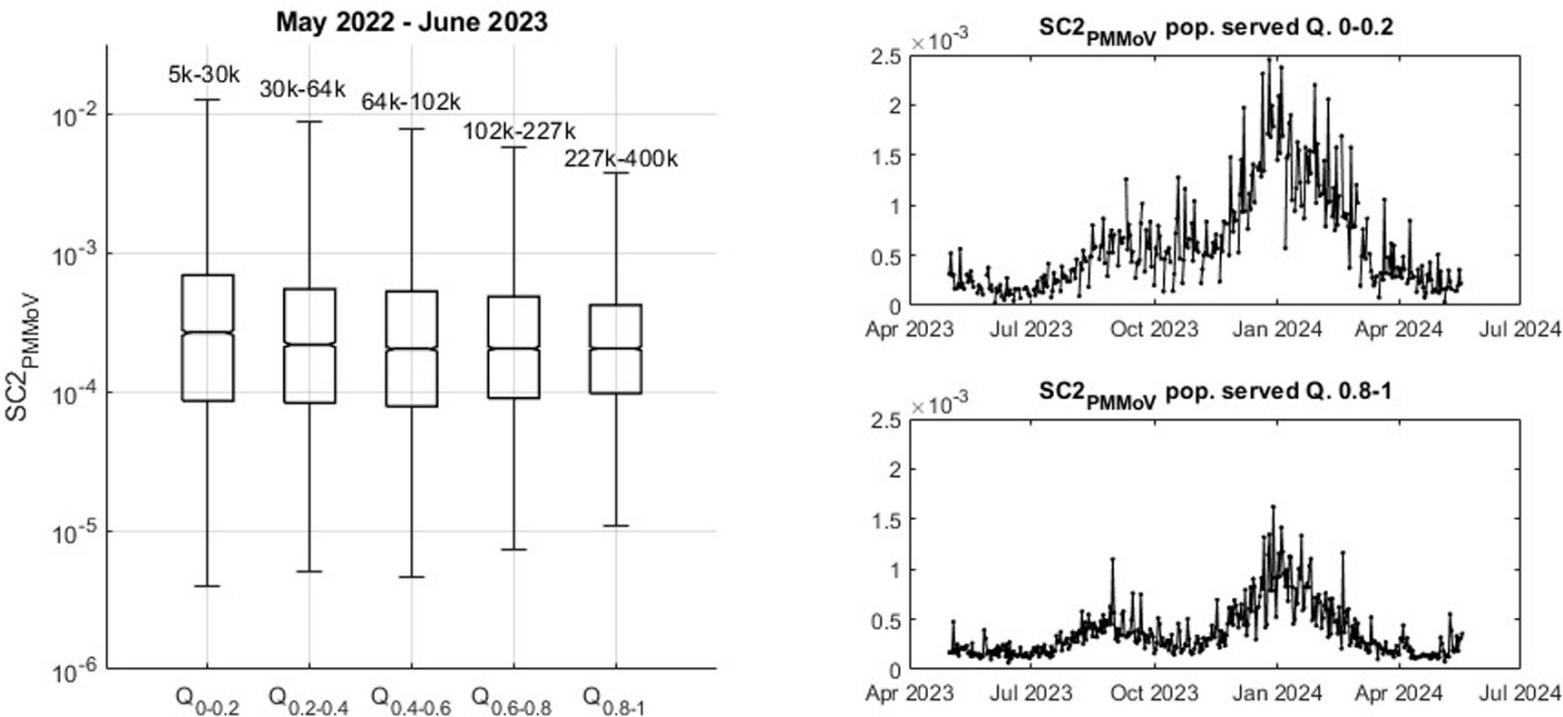
Data variability by quantile grouped data of different WWTPs sizes.

Data dispersion results are listed in Table 4 and visualized in Fig 6. Table 4 describes data mean, median, standard deviation (SD) and interquartile range (IQR) of the partitioned data. The main measures of variability, SD and IQR, are observed to decrease with increasing plant size. This observation is in line with expectations, considering the more stochastic behavior of small plants and the law of large numbers. An intuitive explanation can be provided by considering a case prevalence of 0.1%. In a small plant serving 10,000 people, 10 individuals would be infected. Due to the size of the sewer system and the stochastic shedding behavior of these 10 infected individuals, SC2 concentrations may exhibit significant variability. Conversely, in a large WWTP serving a population of 1 million, 0.1% prevalence would correspond to 1,000 infected individuals. With a significant number of individuals shedding the virus, a more consistent discharge of the virus into the sewer system is likely. These findings align with the results from Nauta *et al*. [32], who performed Monte-Carlo simulations to estimate SC2 concentrations and data variability.

**Table 4:** Data dispersion properties log_10_(SC2) mean, median, standard deviation and interquartile range by plant size.

Quantile	mean log_10_(SC2)	median log_10_(SC2)	SD log_10_(SC2)	IQR log_10_(SC2)
Q_0-0.2_	4.846	4.862	0.569	0.781
Q_0.2–0.4_	4.875	4.877	0.538	0.735
Q_0.4–0.6_	4.858	4.854	0.504	0.687
Q_0.6–0.8_	4.947	4.942	0.477	0.643
Q_0.8–1_	5.001	5.026	0.457	0.612

Fig 6 visualizes the data dispersion as a function of plant size. On the left, a boxplot diagram displays data median, upper and lower quartiles and minimum/maximum values by whiskers. The graph shows consistent decrease of data range and variability with the increase in plant size. On the right of Fig 6 aggregated time-series are graphed, corresponding to Q_0-0.2_ (top, small plants) and Q_0.8–1_ (bottom, big plants). The ordinate axes are scaled equally for comparison.

To test the hypothesis that the partitioned wastewater groups based on plant size originate from statistically different data populations, two-sided Wilcoxon rank sum tests are performed. Four tests are carried out among the five groups between the adjacent groups. All tests reject the null hypothesis (that they stem from the same data population). Therefore, all tests recommend to accept the alternative hypothesis, supporting the hypothesis that there are underlying differences in data variability as a function of plant size.

## 4. Conclusion

The COVID-19 epidemic in the US analyzed form WBE perspective in the time-frame 2023–2024 was characterized by waves, that is periods of high and periods of low viral abundance in wastewater. The epidemic’s burden on the general population was lower–considering that case fatality was 60% lower in the studied timeframe, compared to the same time-frame one-year earlier [2]. This work investigates the SARS-CoV-2 RNA concentration data in US wastewater in 2023–2024. Clinical COVID-19 case reporting was largely discontinued as of March 2024 [2, 33, 34]; hospitalization data reporting was discontinued in early May 2024. In contrast, SARS-CoV-2 wastewater surveillance endeavors (among other pathogens) are now well-established across the US.

The work at hand examines statistical attributes of SARS-CoV-2 RNA and pepper mild mottle RNA concentrations derived from wastewater surveillance. Firstly, temporal features, such as peak timing and CCF lag in the data are analyzed and compared to hospitalization admissions. The observations show that viral RNA abundance in wastewater leads hospitalization admission between 2 and 12 days, in the 10 states with the highest WBE population coverage. Data variability was analyzed and the influence of plant size on data dispersion has been observed, with the results demonstrating that smaller plants are subject to significantly more data variability. By partitioning the data into five batches based on plant size, a decrease in data variability with increased plant size is observed.

In addition to the findings presented in this study, several topics remain open for further investigation. Further research beyond the scope of this study would be needed to investigate the interdependency between vaccination and viral shedding patterns and whether the impact significantly influences WBE data interpretation. Furthermore, as spatial aggregation is underrepresented in current literature, comparing different methods would offer valuable insights.

## Supporting information

S1 File Comparison between national aggregated WBE data. Raw SC2 RNA and PMMoV normalized SC2 RNA concentrations.
(DOCX)

## References

[1] WHO, https://www.who.int/: Date of retrieval 06-01-2024.

[2] CDC, https://covid.cdc.gov/covid-data-tracker/#datatracker-home: Date of retrieval 06-01-2024.

[3] CDC, https://www.cdc.gov/covid/hcp/testing/index.html: Date of retrieval 06-01-2024.

[4] Daughton C. G., "Wastewater surveillance for population-wide Covid-19: The present and future," The Science of the total environment, vol. 736, p. 139631, 2020, doi: 10.1016/j.scitotenv.2020.139631 32474280 PMC7245244

[5] Boehm A. B., Wolfe M. K., White B., Hughes B., and Duong D., "Divergence of wastewater SARS-CoV-2 and reported laboratory-confirmed COVID-19 incident case data coincident with wide-spread availability of at-home COVID-19 antigen tests," PeerJ, vol. 11, e15631, 2023, doi: 10.7717/peerj.15631 37397016 PMC10312197

[6] McClary-Gutierrez J. S. et al., "SARS-CoV-2 Wastewater Surveillance for Public Health Action," Emerging infectious diseases, vol. 27, no. 9, pp. 1–8, 2021, doi: 10.3201/eid2709.210753 34424162 PMC8386792

[7] Wölfel R. et al., "Virological assessment of hospitalized patients with COVID-2019," Nature, vol. 581, no. 7809, pp. 465–469, 2020, doi: 10.1038/s41586-020-2196-x 32235945

[8] Medema G., Heijnen L., Elsinga G., Italiaander R., and Brouwer A., "Presence of SARS-Coronavirus-2 RNA in Sewage and Correlation with Reported COVID-19 Prevalence in the Early Stage of the Epidemic in The Netherlands," Environmental science & technology letters, vol. 7, no. 7, pp. 511–516, 2020, doi: 10.1021/acs.estlett.0c00357 37566285

[9] Ahmed W. et al., "First confirmed detection of SARS-CoV-2 in untreated wastewater in Australia: A proof of concept for the wastewater surveillance of COVID-19 in the community," The Science of the total environment, vol. 728, p. 138764, 2020, doi: 10.1016/j.scitotenv.2020.138764 32387778 PMC7165106

[10] La Rosa G. et al., "First detection of SARS-CoV-2 in untreated wastewaters in Italy," The Science of the total environment, vol. 736, p. 139652, 2020, doi: 10.1016/j.scitotenv.2020.139652 32464333 PMC7245320

[11] Lodder W. and de Roda Husman A. M., "SARS-CoV-2 in wastewater: potential health risk, but also data source," The lancet. Gastroenterology & hepatology, vol. 5, no. 6, pp. 533–534, 2020, doi: 10.1016/S2468-1253(20)30087-X 32246939 PMC7225404

[12] Randazzo W., Truchado P., Cuevas-Ferrando E., Simón P., Allende A., and Sánchez G., "SARS-CoV-2 RNA in wastewater anticipated COVID-19 occurrence in a low prevalence area," Water research, vol. 181, p. 115942, 2020, doi: 10.1016/j.watres.2020.115942 32425251 PMC7229723

[13] Graham K. E. et al., "SARS-CoV-2 RNA in Wastewater Settled Solids Is Associated with COVID-19 Cases in a Large Urban Sewershed," Environmental science & technology, vol. 55, no. 1, pp. 488–498, 2021, doi: 10.1021/acs.est.0c06191 33283515

[14] Peccia J. et al., "Measurement of SARS-CoV-2 RNA in wastewater tracks community infection dynamics," Nature biotechnology, vol. 38, no. 10, pp. 1164–1167, 2020, doi: 10.1038/s41587-020-0684-z 32948856 PMC8325066

[15] Chan E. M. G., Bidwell A., Li Z., Tilmans S., and Boehm A. B., "Public health policy impact evaluation: A potential use case for longitudinal monitoring of viruses in wastewater at small geographic scales," PLOS Water, vol. 3, no. 6, e0000242, 2024, doi: 10.1371/journal.pwat.0000242

[16] Wolfe M. K. et al., "Detection of Hemagglutinin H5 Influenza A Virus Sequence in Municipal Wastewater Solids at Wastewater Treatment Plants with Increases in Influenza A in Spring, 2024," Environmental science & technology letters, 2024, doi: 10.1021/acs.estlett.4c00331

[17] Wolfe M. K. et al., "Wastewater Detection of Emerging Arbovirus Infections: Case Study of Dengue in the United States," Environmental science & technology letters, vol. 11, no. 1, pp. 9–15, 2024, doi: 10.1021/acs.estlett.3c00769

[18] Zulli A. et al., "Prospective study of Candida auris nucleic acids in wastewater solids in 190 wastewater treatment plants in the United States suggests widespread occurrence," mBio, vol. 15, no. 8, e0090824, 2024, doi: 10.1128/mbio.00908-24 39041799 PMC11323724

[19] Kaplan E. H., Wang D., Wang M., Malik A. A., Zulli A., and Peccia J., "Aligning SARS-CoV-2 indicators via an epidemic model: application to hospital admissions and RNA detection in sewage sludge," Health care management science, vol. 24, no. 2, pp. 320–329, 2021, doi: 10.1007/s10729-020-09525-1 33111195 PMC7592141

[20] Galani A. et al., "SARS-CoV-2 wastewater surveillance data can predict hospitalizations and ICU admissions," The Science of the total environment, vol. 804, p. 150151, 2022, doi: 10.1016/j.scitotenv.2021.150151 34623953 PMC8421077

[21] Schenk H. et al., "Prediction of hospitalisations based on wastewater-based SARS-CoV-2 epidemiology," The Science of the total environment, vol. 873, p. 162149, 2023, doi: 10.1016/j.scitotenv.2023.162149 36773921 PMC9911153

[22] Li X. et al., "Wastewater-based epidemiology predicts COVID-19-induced weekly new hospital admissions in over 150 USA counties," Nature communications, vol. 14, no. 1, p. 4548, 2023, doi: 10.1038/s41467-023-40305-x 37507407 PMC10382499

[23] Bibby K., Bivins A., Wu Z., and North D., "Making waves: Plausible lead time for wastewater based epidemiology as an early warning system for COVID-19," Water research, vol. 202, p. 117438, 2021, doi: 10.1016/j.watres.2021.117438 34333296 PMC8274973

[24] Olesen S. W., Imakaev M., and Duvallet C., "Making waves: Defining the lead time of wastewater-based epidemiology for COVID-19," Water research, vol. 202, p. 117433, 2021, doi: 10.1016/j.watres.2021.117433 34304074 PMC8282235

[25] Boehm A. B. et al., "Human viral nucleic acids concentrations in wastewater solids from Central and Coastal California USA," Scientific data, vol. 10, no. 1, p. 396, 2023, doi: 10.1038/s41597-023-02297-7 37349355 PMC10287720

[26] WastewaterSCAN, https://data.wastewaterscan.org/: Date of retrieval 06-01-2024.

[27] Boehm A. B. et al., "Human pathogen nucleic acids in wastewater solids from 191 wastewater treatment plants in the United States," Scientific data, vol. 11, no. 1, p. 1141, 2024, doi: 10.1038/s41597-024-03969-8 39420189 PMC11487133

[28] Arts P. J. et al., "Longitudinal and quantitative fecal shedding dynamics of SARS-CoV-2, pepper mild mottle virus, and crAssphage," mSphere, vol. 8, no. 4, e0013223, 2023, doi: 10.1128/msphere.00132-23 37338211 PMC10506459

[29] Wolfe M. K. et al., "Scaling of SARS-CoV-2 RNA in Settled Solids from Multiple Wastewater Treatment Plants to Compare Incidence Rates of Laboratory-Confirmed COVID-19 in Their Sewersheds," Environmental science & technology letters, vol. 8, no. 5, pp. 398–404, 2021, doi: 10.1021/acs.estlett.1c00184 37566351

[30] Duvallet C. et al., "Nationwide Trends in COVID-19 Cases and SARS-CoV-2 RNA Wastewater Concentrations in the United States," ACS ES&T water, vol. 2, no. 11, pp. 1899–1909, 2022, doi: 10.1021/acsestwater.1c00434 36380771 PMC9092192

[31] Zulli A., Varkila M. R. J., Parsonnet J., Wolfe M. K., and Boehm A. B., "Observations of Respiratory Syncytial Virus (RSV) Nucleic Acids in Wastewater Solids Across the United States in the 2022–2023 Season: Relationships with RSV Infection Positivity and Hospitalization Rates," ACS ES&T water, vol. 4, no. 4, pp. 1657–1667, 2024, doi: 10.1021/acsestwater.3c00725 38633368 PMC11019535

[32] Nauta M. et al., "Early detection of local SARS-CoV-2 outbreaks by wastewater surveillance: a feasibility study," Epidemiology and infection, vol. 151, e28, 2023, doi: 10.1017/S0950268823000146 36722251 PMC9990400

[33] John Hopkins University & Medicine, https://coronavirus.jhu.edu/map.html. Date of retrieval 31-07-2024.

[34] New York Times, https://www.nytimes.com/interactive/2021/us/covid-cases.html. Date of retrieval 31-07-2024.

