# Supplementary material for: SARS-CoV-2 surveillance in US wastewater: Leading indicators and data variability analysis in 2023–2024
Source: PLoS One. 2024 Nov 18;19(11):e0313927. doi: 10.1371/journal.pone.0313927 (PMC11573121; doi:10.1371/journal.pone.0313927)
Supplement: S1 File — Raw SC2 RNA and PMMoV normalized SC2 RNA concentrations. (DOCX) [file pone.0313927.s001.docx]

Supporting information

The analyses outlined in the paper have been performed on both raw SC2 concentrations and PMMoV normalized data. We present the results of the PMMoV normalized analyses, considering it is a standard procedure in the field. The conclusions remain unchanged whether PMMoV normalization is applied or not.

The figure below shows national aggregated raw SC2 data and PMMoV normalized SC2 data. It can be seen, that the shape of the data is in close agreement.


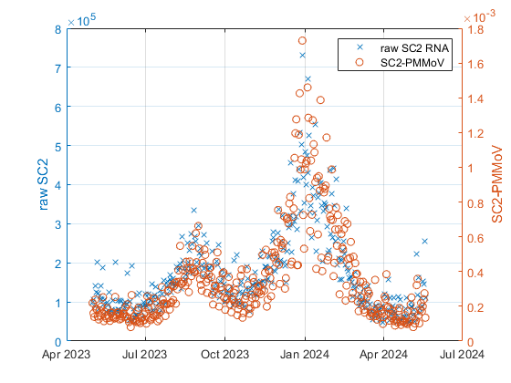


S 1: Comparison between national aggregated WBE data: Raw SC2 RNA and PMMoV normalized SC2 RNA concentrations.
